# Telehealth Family Psychoeducation for Major Depressive Disorder: A Protocol for Intervention Co-Design and Feasibility Study

**DOI:** 10.3390/nursrep15100364

**Published:** 2025-10-11

**Authors:** Obumneke Obieche, Jing-Yu (Benjamin) Tan, Sita Sharma, Daniel Bressington, Tao Wang

**Affiliations:** 1 School of Nursing and Midwifery, University of Southern Queensland, Ipswich, QLD 4305, Australia; benjamin.tan@unisq.edu.au (J.-Y.T.); sita.sharma@unisq.edu.au (S.S.); daniel.bressington@cmu.ac.th (D.B.); 2 Centre for Health Research, University of Southern Queensland, Springfield, QLD 4300, Australia; 3 Faculty of Nursing, Chiang Mai University, Chiang Mai 50200, Thailand

**Keywords:** co-design, family psychoeducation, major depressive disorder, psychosocial support, telehealth

## Abstract

**Background/Objectives:** Limited access to mental health services contributes to poorer outcomes among individuals with mental health conditions, including major depressive disorder (MDD). Nurse-led interventions serve as a strategic model of care to improve mental health service delivery and enhance patient outcomes. This project aims to co-design a nurse-led telehealth family psychoeducation (FPE) for MDD and primarily assess its feasibility by evaluating the recruitment and retention rates. **Methods:** A multi-methods study encompassing a co-design phase (Study Phase 1) and a feasibility study (Study Phase 2). Study Phase 1 will involve semi-structured interviews with individuals with MDD and their families or significant others, as well as surveys and focus groups with mental health professionals to develop telehealth FPE for MDD. Study Phase 2 will evaluate the feasibility and acceptability of the intervention, which comprises three biweekly FPE sessions and a six-week follow-up with patient–family dyads using a single-group pre-post design. The primary outcomes comprise the feasibility and acceptability of intervention. Exploratory secondary outcomes include personal recovery, medication necessity beliefs and concerns, antidepressant adherence, and depression severity, measured at baseline, immediately post-intervention, and at 6-week follow-up using validated measures. Data analysis will primarily involve descriptive statistics and thematic analysis. The TIDieR checklist will be followed in reporting the intervention development. **Conclusions:** Findings from the proposed study will inform the design and protocol for a future randomised trial of telehealth FPE for improving clinical and non-clinical outcomes in MDD. The feasibility study was prospectively registered with the ClinicalTrial.gov on 8 June 2025 (NCT07014241).

## 1. Introduction

Major depressive disorder (MDD) is a highly prevalent mental disorder, affecting an estimated 5% of adults globally [1]. It is characterised by significant emotional, cognitive, and physical symptoms leading to considerable distress or impairment in psychosocial or occupational functioning [2]. The impact of MDD often extends beyond the person experiencing depressive symptoms. It indirectly contributes to poorer economic outcomes and quality of life for family members or caregivers of adults with MDD [3].

Clinical practice guidelines emphasise the importance of a comprehensive approach to managing MDD [4,5]. Specifically, psychoeducation, psychological therapy, and lifestyle modifications are recommended as foundational interventions that should be implemented from the outset for functional recovery from MDD, while antidepressant medications remain the mainstay pharmacological treatment for alleviating acute depressive symptoms [5]. Research indicates that psychoeducation as an adjunct to antidepressant treatment improves clinical and psychosocial outcomes for patients with MDD compared to the control group [6].

Psychoeducation is a structured psychosocial intervention that helps individuals and their families gain knowledge and skills to manage illness and work collaboratively with healthcare professionals for better overall outcomes [7,8]. Family involvement in psychoeducation empowers families with understanding and skills on how to support the recovery of their loved ones with a mental illness [9], as well as help in addressing misconceptions about mental illness within families [10]. Findings from a meta-analytical study showed that family psychoeducation (FPE) resulted in a statistically significant improvement in depressive symptoms among adult patients compared to the control group at short and long term [11]. Similarly, results from a randomised controlled trial (RCT) indicate that brief multifamily psychoeducation for family members of patients with MDD decreased depressive symptoms among family members and enhanced family functioning in comparison to a control group, which received a single counselling session [12].

Despite evidence supporting FPE for MDD [11,13], research on this intervention remains limited [11]. Some existing studies involved only patients [14,15] or patients’ family members or caregivers [12,16]. These psychoeducational approaches may inherently limit the benefits of shared learning and support reinforcement within a family context, given that studies have demonstrated significant improvements in family support following psychoeducational interventions involving both patients and their families [17]. Additionally, most prior studies on FPE for MDD [18,19,20] did not report patient and public involvement (PPI) at any stage, including during study design, data interpretation, implementation, or evaluation of study impact. Specifically, co-design methodologies involving mental health service users, their families or caregivers, and healthcare providers have contributed to the development of effective initiatives [21], as well as improved care, care processes, and collaboration between mental health service users and providers [22]. Given the growing interest in PPI in healthcare [23] and the increasing demand for better reporting of PPI activities [24], research efforts aimed at improving health researchers’ understanding of PPI and fostering meaningful engagement among stakeholders [25,26] have been well-documented in the current literature.

Ensuring that adjunctive interventions are easily accessible to their intended recipients through effective implementation strategies is essential for their adoption and the evaluation of their impact [27]. Telehealth, which involves the digital communication technologies for healthcare delivery, has been recognised as a transformative approach to enhance access to healthcare and interventions [28]. To the best of our knowledge, none of the previous studies that have investigated psychoeducational interventions in patients with MDD and their families or significant others utilised the telehealth method. A study conducted in the United States utilising data from a representative sample of the population found that telehealth is a promising method for accessing mental health care, particularly for individuals diagnosed with depression [29]. Although studies have highlighted challenges associated with the use of telehealth, such as technological and therapeutic relationship issues [30,31], limited access to digital technology and digital literacy and diminished physical skills to operate devices [32], as well as concerns regarding privacy, regulatory policies, and therapeutic relationships [31], telehealth remains an integral part of the contemporary mental health system [33].

The sustained utilisation of telehealth is attributed to its demonstrated cost-effectiveness, high patient satisfaction, and its capacity to provide effective care [34]. Furthermore, research shows that telehealth is widely embraced due to its ease of use and convenience [29,34], as well as its potential to improve access to healthcare, particularly for individuals residing in geographically remote areas or vulnerable populations [35]. Consequently, telehealth offers a practical and convenient approach to overcoming some of the barriers to accessing mental health care and services [36]. Although the COVID-19 pandemic and related social distancing measures led to the expansion of telehealth mental health services globally [37], it remains essential to evaluate how feasible it is to integrate telehealth FPE into routine psychosocial support for individuals with MDD after the pandemic.

The proposed study, therefore, aims to co-design FPE, known as Supportive Program for Advancing Recovery, Knowledge, and Empowerment in Depression (SPARKED), and to assess the feasibility and acceptability of SPARKED delivered through telehealth among individuals with MDD and their families. The primary objectives of the study are as follows:

1. To develop SPARKED through the involvement of patients, patients’ families, and mental health professionals.
2. To determine the feasibility of SPARKED through assessing recruitment and retention rates.
3. To determine the feasibility of outcome measure completion and return rates.
4. To determine the acceptability of SPARKED at pre-intervention, during intervention delivery, and post-intervention.

The secondary objective of the study is to assess the preliminary effects of SPARKED on personal recovery, depression severity, antidepressant adherence, and necessity beliefs and concerns about antidepressants among individuals with MDD.

## 2. Materials and Methods

### 2.1. Overall Study Design

**Figure 1:**
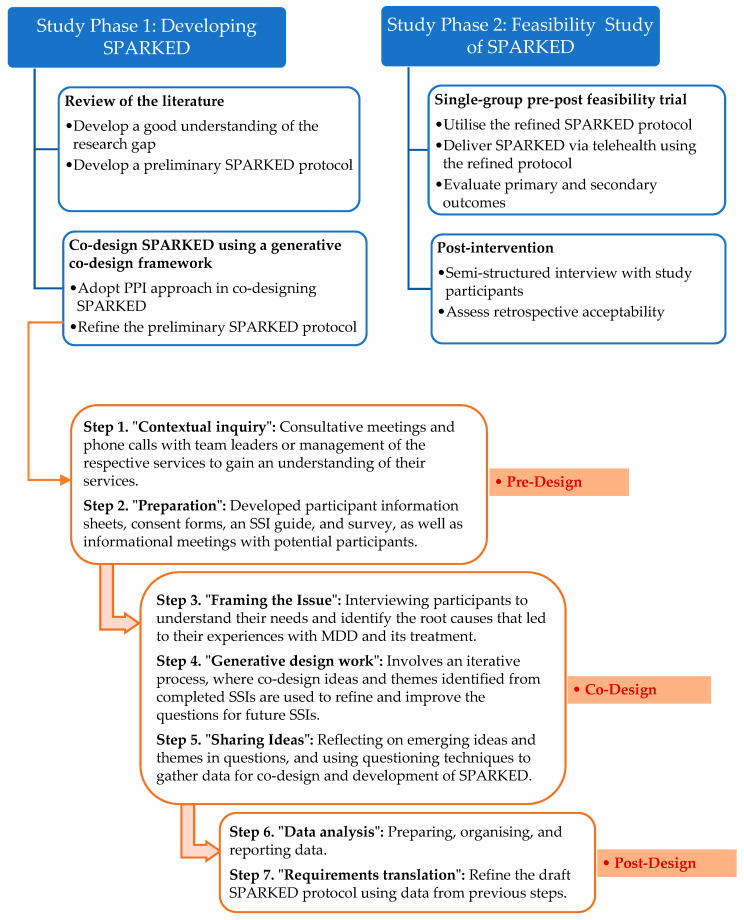
Overall study design informed by the MRC framework [38] and generative co-design framework [39] (p. 5).

A multi-method study design will be used. This study will adopt the updated Medical Research Council (MRC) framework for developing and evaluating a complex intervention [38], while the co-design strategies will align with the Generative Co-design framework [39]. The MRC framework outlines four phases of complex intervention research: the development (Phase 1), assessment of feasibility and evaluation design (Phase 2), evaluation of the intervention (Phase 3), and implementation (Phase 4) [38]. This study will involve the first two phases within the MRC framework to develop SPARKED (Study Phase 1) and evaluate its feasibility (Study Phase 2). The generative co-design framework [39] identifies three main phases guiding the PPI process in health innovation: predesign, co-design, and post-design phases, which encompass seven distinct steps (Figure 1).

To target some key factors hindering better outcomes in MDD [40], this study will utilise the Necessity–Concerns Framework [41]. This framework will help tailor the study intervention to address the beliefs, misconceptions, and concerns that participants may have about MDD, its treatment, or services available for users. The reporting of this research will follow the Template for Intervention Description and Replication (TIDieR) guidelines [42]. The sub-studies and key research activities planned for Study Phases 1 and 2 are outlined in Figure 1.

### 2.2. Study Phase 1: Developing SPARKED

#### 2.2.1. Study Design

Study Phase 1 will employ a multi-method design to support the development of SPARKED. This multi-method approach involves reviewing the literature to identify evidence on FPE for MDD and three-layered co-design strategies, including

1. Semi-structured interviews (SSIs) with patients diagnosed with MDD and their families;
2. Surveys of mental health professionals;
3. Focus group of mental health professionals.

Through a comprehensive systematic review, which is yet to be published, the study team found that research on FPE involving individuals with MDD and their family members or significant others is limited. Additionally, the study team developed a preliminary SPARKED guide (Supplementary Material, Table S1), which will be refined using data gathered through the co-design strategies.

#### 2.2.2. Study Setting

Recruitment of participants for the SSIs will take place through study flyers displayed at five primary care settings, including a primary healthcare clinic, two psychological services, a non-profit and charitable peer-led association, and a community pharmacy, all located in Darwin, Northern Territory, Australia. In addition, recruitment can be via a referral pathway informed by general practitioners (GPs) or nurse practitioners at the primary healthcare clinic. The SSIs will be held either at the study sites or in meeting rooms within community halls, depending on participants’ preferences. The online surveys and focus groups will involve mental health professionals recruited from public mental health services in the Northern Territory.

#### 2.2.3. Semi-Structured Interviews with Patients and Their Family Members

##### Sample and Sampling

This sub-study will involve SSIs with adult patients and their family members or significant others. In defining the inclusion and exclusion criteria for this study, an adult is considered a person who is 18 years or older [43].

The inclusion criteria are as follows: (i) an adult diagnosed with MDD who has an adult family member, carer, or significant other involved in supporting their recovery; (ii) both the adult with MDD and their family member, carer, or significant other voluntarily consent to participate in the study; (iii) both the adult with MDD and their family member, carer, or significant other have basic English language skills, allowing them to engage in the study. Interested individuals who meet the inclusion criteria will be enrolled consecutively according to their availability.

Exclusion criteria include (i) an individual with MDD, a family member, carer, or significant other under 18 years, (ii) an adult with MDD whose family member, carer, or significant other cannot participate in the study, (iii) an adult with MDD, family member, carer, or significant other who has a serious mental or physical health condition or cognitive impairment that may affect the quality of their participation in the study.

Strategies for recruiting representative participants for this sub-study include using a multi-channel approach; providing detailed study information to potential participants’ referrers at the study sites; using clear and culturally appropriate language on the flyers that succinctly describe the study goals and process; involving individuals with lived experience of mental health issues in reviewing the study flyers; ensuring respectful and trusting relationships with potential participants, and offering financial compensation for time and transportation costs.

The sample size for this sub-study will be determined based on data saturation, which typically occurs within 9 to 17 interviews [44]. Considering resource availability and the scope of this sub-study’s objectives, ten SSIs are planned. However, the actual sample size will be determined by data saturation. Data saturation will be assessed through an iterative process of collecting and analysing data simultaneously to identify when no new themes emerge, indicating that data saturation has been reached. In applying the data saturation technique, consideration will be given to the quality of the data collected that pertains to the study’s objectives.

##### Study Procedure

Interested individuals can contact the study team using the contact details on the flyer to express their interest in the study or request study information. Following the expression of interest, each patient–family member dyad (i.e., an individual diagnosed with MDD and their family member or significant other) will attend a face-to-face meeting, where they will be provided with further study details and a participant information sheet. Written consent will be obtained before enrolling each participant.

Face-to-face SSIs will be conducted with each patient–family member dyad using an SSI guide developed by the study team, based on the study objectives (Supplementary Material, Table S2). The guide includes 11 opening questions and related probing questions. Patients will be asked all 11 opening questions, whereas their family members will be asked all except the first two, which explore how long the patient has been diagnosed with MDD and the treatments received. Examples of the questions are as follows: “Can you tell me the type of treatment for depression you have received?” and “What kind of information would you like healthcare professionals involved in depression care to provide to patients living with depression and their families?”

In line with the post-design phase within the generative co-design framework [39], data collected from each SSI will be analysed to help refine the SSI questions that will be asked in the next SSI. Furthermore, the research team will disseminate generalised findings from the co-design SSIs to participants who have expressed interest in receiving the result summary through their preferred mode of communication.

A single-family format will be adopted to reduce the risk of privacy breaches. Each SSI will last between 45 and 60 minutes and will be audio-recorded to ensure accurate data collection. Using a purposeful questionnaire, basic demographic information will be solicited from the study participants.

The research team will undertake several quality assurance activities to ensure the quality of the research process and output. The interviewer (OO) will maintain neutrality during the interviews, regularly reflect on personal views and assumptions, and keep field notes. The team will engage in continuous reflection on its role at each stage of the research. This will be achieved through regular research team meetings that facilitate reflection on the research process, data analysis, and quality assessment, thereby ensuring reflexivity and best practices. Additionally, member checking, where the study team shares preliminary study findings with participants and seeks their feedback, will be employed.

##### Data Analysis

Data analysis will include descriptive statistics and thematic analysis to identify themes and patterns within a dataset. The thematic analysis will follow the six-step phases described by Braun and Clarke [45]. The thematic analysis will involve an iterative approach to identify codes representing specific ideas within data segments, organise multiple codes in a dataset into themes, review and refine themes, and define the generated themes. The identified themes will be interpreted to provide an overall understanding of the findings.

#### 2.2.4. Surveys of Mental Health Professionals

##### Sample and Sampling

This sub-study will involve an anonymous online survey of mental health professionals. The inclusion criteria are as follows: (i) a mental health professional with either a permanent or fixed-term employment contract, and (ii) voluntarily consents to participate in the study. The exclusion criterion is a mental health professional who is employed as a casual employee or agency staff member within the mental health service. Due to frequent job transitions, casual employment contracts may limit knowledge of the issues this sub-study aims to explore. The key sections of the survey, consisting of six items, will collect data relevant to the co-design of SPARKED. A sample size of 30 is determined using a 5:1 respondent-to-item ratio [46]. Participants will be recruited through convenience sampling, with survey links distributed via email.

##### Study Procedure

The survey was developed based on the study’s objective and relevant literature (Supplementary Material, Table S3). It comprises three sections: The first collects basic professional information; the second includes three questions that examine respondents’ perspectives on the information frequently sought by individuals with MDD and their families; and the third gathers opinions on telehealth FPE for MDD. The second and third sections contain open-ended questions, such as “What information do patients with MDD and their family members or significant others seek from health professionals involved in depression care or treatment?” and “What do you consider essential for effective telehealth family psychoeducation for MDD?”, respectively. The survey was pilot tested to identify potential issues with question clarity. Data from the pilot test will not be included in the study results because respondents’ eligibility was not assessed.

To recruit study participants, an email invitation to participate in the survey will be sent to mental health professionals. The invitation email will provide details about the study, including a participant information sheet, the study team’s contact details, and links to a consent form and the survey. To proceed with completing the survey, participants must acknowledge that they have read the consent form and consented to participate.

##### Data Analysis

Data will be separated into quantitative and textual components. Using a frequency table, descriptive statistics will summarise both numerical and categorical data, including the number of respondents, the modal number of years of mental health practice, and proportions of different professions, highest qualifications, and genders in the sample. Content analysis will be used to examine textual data to identify patterns and themes within the responses. The extent and nature of missing data will be reported.

#### 2.2.5. Focus Group of Mental Health Professionals

##### Sample and Sampling

Participants will be recruited from the population of mental health professionals who received an invitation email to participate in the online survey (as described above). The email and the final survey page will request expressions of interest in a focus group discussion (FGD) to co-design telehealth FPE for MDD. Written consent will be obtained from each participant prior to enrolment. Given that the online survey is anonymous, the study team will not be able to confirm whether the focus group participants also completed the survey.

The FGD will involve a sample of 10 mental health professionals, grouped into two focus groups. This sample size is based on studies showing that two to three focus groups capture at least 80% of themes on a topic in a study with a homogeneous population using a semi-structured guide [47], while a small focus group of between four and six individuals is appropriate for health research [48].

##### Study Procedure

Eligible participants will be invited to express their interest in the focus group by replying with ‘Yes to a Focus Group’ to the study invitation email. Following an expression of interest from a potential participant, they will be contacted to provide more information about the focus group and explain the consent process. Reminder emails will be sent to mental health professionals to enhance the chances of achieving the target sample size.

The focus groups will be conducted virtually via the Zoom platform; however, participants’ preferences for alternative platforms will be duly considered. Meeting links will be emailed to participants. The FGDs will be facilitated using a guide developed based on the results of previous co-design strategies in this study and existing literature. To ensure accurate data collection, the FGDs will be audio-recorded, and field notes will be maintained. The implementation of the focus groups will follow established practical guidance for conducting virtual focus groups [49]. All quality assurance activities outlined in Section Study Procedure will be applied at all stages of this sub-study.

##### Data Analysis

Data analysis will follow the same process as outlined for the SSI data in Section Data Analysis. Codes and themes generated from data analysis will further inform the development of the SPARKED guide, which will be used in Study Phase 2.

### 2.3. Study Phase 2: Feasibility Study of SPARKED

#### 2.3.1. Study Design

A single-group, pre-post feasibility trial with an embedded qualitative evaluation.

#### 2.3.2. Study Setting

This study will be conducted in three general practice clinics located in a regional area in the Northern Territory, Australia.

#### 2.3.3. Participants and Sample Size

Patient–family member dyads will be recruited through study flyers or a GP- or nurse practitioner-informed referral pathway.

Inclusion criteria are as follows: (i) an adult (aged 18 years or older) diagnosed with MDD who has been prescribed at least one antidepressant medication for treating the disorder and can provide a formal record of a current prescription, (ii) an adult with MDD who does not have any other serious mental or physical illness, (iii) an adult with MDD who has an adult family member, a carer, or significant other supporting themat home, and (iv) both the adult with MDD and their support person consent to participate in the study.

Exclusion criteria are: (i) an adult with MDD whose family member, carer or significant other cannot participate in the study, (ii) an adult family member of an adult with MDD who declines to participate, (iii) an adult with MDD identified with high suicide risk or current suicidal ideation using a suicide risk assessment tool [50], and (iv) an adult with MDD or a family member with a serious mental or physical health condition, cognitive impairment, or hearing impairment without a hearing aid. These health conditions may affect an individual’s ability to participate effectively in telehealth sessions.

The SS-PROGRESS WebApp (SS-PROGRESS) [51] was used to calculate the sample size required for the binary feasibility outcomes for estimation and hypothesis testing of pro-gression criteria. The 1-sample 1-tailed binomial exact test tests the null hypothesis that the true feasibility outcome is not greater than the upper “RED” stop limit [52]. Assuming an alpha of 0.05 and a beta of 0.2, we applied a minimum value of acceptable feasibility of 34% (indicating the smallest percentage for an outcome, which suggests that the main study would be feasible) and an expected value of 70% for each feasibility objective, resulting in a minimum sample size of 13.

#### 2.3.4. Study Intervention

The study intervention, SPARKED, is a brief FPE that will be delivered via telehealth using the SPARKED guide developed in Study Phase 1. The guide will contain information on predetermined topics organised into modules for each psychoeducational session, which will be conducted biweekly at Weeks 2, 4, and 6 from enrolment. A six-week follow-up at Week 12 will primarily focus on assessing the retrospective acceptability of SPARKED. The key features of the SPARKED protocol are shown in Table 1.

The sessions will be tailored to enhance participants’ understanding of MDD and its treatment, coping strategies, self-efficacy skills, and early signs of relapse, empowering them to take an active role in their treatment decision-making. Only one member of the research team will facilitate all SPARKED sessions to ensure consistency throughout the sessions. During each session, evidence-based information will be provided, and participants will be encouraged to seek further discussion and clarification as needed.

#### 2.3.5. Study Procedure

Patients with MDD who are interested in the study can contact the research team using the contact details on the study flyers for more information or to express interest. If the participants meet the eligibility criteria, a face-to-face meeting will be scheduled with each single-family dyad to provide them with detailed information about the study, explain the consent process, and distribute participant information sheets. The meetings will be held either at the primary healthcare setting where participants are recruited or in meeting rooms within community halls, depending on participants’ preferences. In addition, suicide risk assessments of individuals with MDD will be conducted using the suicide risk screening tool [50]. If imminent risk is identified, individuals will be supported with a stepped-up care referral. Written consent will be obtained from each eligible participant before they are enrolled in the study.

All baseline assessments will be conducted immediately after enrolment. Basic sociodemographic data, along with medical and medication histories, will be collected from individuals with MDD. Similarly, sociodemographic information will be obtained from the participants’ family members or significant others. Upon completion of the pre-intervention assessments, the initial SPARKED telehealth session will be scheduled. Participants will select their preferred telehealth method (videoconferencing or telephone call) based on their individual circumstances, as well as a secure environment for each session. Subsequent sessions will be scheduled at the end of each session. The Zoom platform will be utilised for videoconferencing; however, participants’ preferences for an alternative platform will be considered. Reminder messages or phone calls, based on participants’ individual preferences, will be used to remind them of their upcoming session. To reduce the risk of incomplete intervention, the family member or significant other supporting the individual with MDD will remain consistent throughout the study.

A contingency plan to prevent dropout includes offering flexible session scheduling, allowing participants to choose their preferred telehealth modality, and providing phone credits for each session, along with modest tokens to compensate for their time. Participants will be advised that they can reschedule a session if necessary. Subsequently, a new session will be organised. In addition, reminder phone messages or calls, according to participants’ preferences, will be made one to two days prior to each scheduled session.

Participants will complete the second and third sets of outcome measurements immediately after the intervention (Week 6) and at follow-up (Week 12), respectively. Measures can be completed on hard copies and mailed to the research team using prepaid envelopes provided by the team or completed online. Additionally, SSIs will be conducted with each patient–family member dyad at follow-up to collect feedback on the intervention. All FPE sessions and the SSIs will be audio recorded with participants’ consent, allowing the research team to conduct checks on adherence to the intervention guide and protocol. Field notes will be maintained at all sessions.

#### 2.3.6. Treatment Integrity

The first author (OO) will deliver the study intervention during all sessions. OO is a nursing doctoral candidate with extensive training in dialectical behavioural therapy. As a research team member, OO has an in-depth understanding of the intervention protocol. To assess adherence to the SPARKED protocol, audio recordings will be independently assessed by authors J-YT, SS, DB, and TW. This assessment will focus on the content and its delivery, as well as the duration and participant engagement.

#### 2.3.7. Outcome Measures

##### Primary Outcomes

The primary outcomes are the feasibility of delivering SPARKED through telehealth and its acceptability among study participants. The feasibility-related outcomes include:

1. Recruitment rate: The main feasibility outcome of this study is the recruitment rate, which was referred to in the sample size calculation. The recruitment rate is defined as the percentage of eligible participant dyads who consented to participate in the study. The observed proportion (E) will be described based on the following pre-defined thresholds: the trial is feasible (if E ≥ 70%); the trial may be feasible, so proceed with caution (if E falls between 33 and 70%); and the trial is unfeasible to proceed (E ≤ 33%).
2. Retention rate: This is defined as the percentage of participants who remain in the study throughout the entire study period, from enrolment to follow-up (Week 12). The observed proportion (E) will be described in the same way as the recruitment rate.
3. Outcome measure return rate: Defined as the percentage of participants who return filled-out self-report measures at enrolment, immediately post-intervention (Week 6), and at follow-up (Week 12).
4. Outcome measure completion rate: Defined as the percentage of participants who successfully complete each secondary outcome measure at enrolment, immediately post-intervention (Week 6), and at follow-up (Week 12).

The acceptability-related outcomes will be assessed at three time points: pre-intervention (prospective acceptability), during intervention delivery (concurrent acceptability), and post-intervention (retrospective acceptability), in line with the theoretical concept of acceptability of a healthcare intervention postulated by Sekhon et al. [53].

1. Prospective acceptability-related outcome: The number of eligible participants who consented to study participation.
2. Concurrent acceptability-related outcome: The number of SPARKED sessions attended and the reasons for dropout during the study period, from enrolment to the follow-up (Week 12).
3. Retrospective acceptability-related outcome: Feedback on SPARKED delivered via telehealth will be assessed through SSIs using an SSI guide (Supplementary Material, Table S4) at follow-up (Week 12).

##### Secondary Outcomes

Four secondary outcomes will be measured at baseline, immediately after the intervention, and at the six-week follow-up using validated instruments. These outcomes are exploratory and will not inform a future RCT.

1. Personal recovery among patients: This outcome refers to subjective recovery, which will be assessed using the Recovery Assessment Scale-Domains and Stages (RAS-DS), a 38-item self-rated measure of personal recovery [54]. Studies have shown that RAS-DS is relevant to recovery [55] and demonstrates good reliability and validity, reflecting mental health recovery [56].
2. Severity of depressive symptoms among patients: This outcome will be measured using the Patient Health Questionnaire-9 (PHQ-9), a nine-item self-administered tool [57]. The choice of PHQ-9 is based on evidence supporting its reliability and validity for assessing depression severity [58].
3. Adherence to antidepressant medication among patients: Antidepressant adherence will be assessed using the Medication Adherence Report Scale-5 (MARS-5, ©Professor Rob Horne), a five-item self-administered instrument that measures both intentional and unintentional nonadherence behaviours [59]. Studies show that MARS-5 is a reliable and valid adherence measure [59,60]. Besides, the wording in MARS-5 is non-judgmental, which may reduce the tendency to underreport medication nonadherence due to social desirability [59].
4. Medication Necessity Beliefs and Concerns among patients: Patients’ necessity beliefs and concerns about antidepressants will be assessed using the Beliefs about Medicines Questionnaire (BMQ)-Specific 11 (BMQ-Specific 11, © Professor Rob Horne) [61]. This instrument comprises two scales: Specific-Necessity and Specific-Concerns [61] and has been reported as an effective tool for identifying patients at risk of medication nonadherence [62], which may be useful in developing interventions to address nonadherence.

#### 2.3.8. Risk Mitigation Plan and Data Management

Risk mitigation plan for managing participant distress includes suicide risk screening (as previously mentioned in Section 2.3.5) and collaborative development of personalised safety plans [63] during the recruitment process, in addition to monitoring adverse effects. The safety plan will outline a participant’s warning signs of an imminent crisis, distress-coping strategies, and available helpful resources, including supportive social networks. It will also include contact details for the participants’ primary healthcare provider (if provided by the participant), the local mental health helpline and emergency services for stepped-up care. Escalation of care will be prioritised once distress is reported or observed, triggering the use of the safety plan. The interventionist will employ empathetic and therapeutic communication skills to minimise further distress and maintain a trusting relationship with the participants.

All research materials will be stored in a lockable cabinet with restricted access. Electronic data will be password-protected and securely stored on the institutional OneDrive, accessible solely to members of the primary research team. Participants will be informed about the importance of utilising private space during SPARKED sessions and post-intervention SSIs. To ensure access to a private internet source during SPARKED sessions and SSIs, each dyadic participant will be offered 2 GB of data per session. Serial numbers will substitute participants’ details to ensure anonymity, and all research outputs will be devoid of any personally identifiable information relating to participants.

In accordance with the International Council for Harmonisation Guideline for Good Clinical Practice [64] and the ethical approval for this research, the research team will conduct ongoing safety assessments, including the monitoring and reporting of adverse effects to the relevant ethics committees. Furthermore, measures implemented to address these adverse effects will be duly reported to the ethics committees.

#### 2.3.9. Quality Assurance and Data Analysis

The study team will undertake quality assurance activities as previously outlined in Section 2.2.3. Data analysis will include both descriptive and inferential statistics.

Outcomes related to feasibility and acceptability (both prospective and concurrent) will be presented as either absolute or relative counts. Data collected through the SSIs (retrospective acceptability data) will undergo thematic analysis following the same procedure outlined in Study Phase 1. Codes and themes identified through this process will be used to refine the SPARKED guide and protocol for future large-scale trials. Conversely, data from each outcome measure will be prepared according to the developer’s instructions. Missing data will be handled using the pairwise deletion method, where missing data are omitted on an analysis-by-analysis basis. Given the small sample size, the Friedman test (significance level of 0.05) will be used to assess whether there are significant differences in the means of outcome measurements across the three measurement time points. The results of this analysis are entirely exploratory research findings.

## 3. Planned Reporting of Results

The TIDieR checklist [42] will be followed in reporting the development of SPARKED, ensuring thorough reporting and replicability of the intervention. The report will include detailed descriptions of the intervention elements, such as materials, procedures, interventionists, mode of delivery, the number of SPARKED sessions, and any modifications implemented.

Triangulation in this research will involve the combination of key findings from co-design SSIs with participants who have MDD and their family members, surveys of mental health professionals, and focus group discussions with mental health professionals. This approach will enable an in-depth understanding of diverse perspectives on telehealth family psychoeducational intervention for MDD. Themes and codes derived from qualitative data will be systematically tabulated, while the contextual narratives will be presented.

Secondary outcomes of the feasibility study of SPARKED will be tabulated across different measurement timepoints. Results on recruitment, retention, and study completion rates will guide the timeline and study duration of a future RCT within the same context. Likewise, feedback on SPARKED (retrospective acceptability) will be used to improve the SPARKED protocol for a subsequent RCT.

## 4. Discussion

This protocol details the co-design strategies and feasibility assessment of SPARKED, a family psychoeducational intervention for MDD, delivered through telehealth. This study will also examine the preliminary effects of SPARKED on both clinically related outcomes (antidepressant adherence and depression severity) and non-clinically related outcomes (medication necessity beliefs and concerns, as well as personal recovery). The results on these outcomes will indicate the direction of an effect and will not be used to determine an effect size for a sample size calculation in a subsequent fully powered trial.

Major depressive disorder is a debilitating mental health condition that significantly impacts an individual’s life and, consequently, their family [65], thereby contributing to the burden of MDD. The impact of MDD is further compounded, given that residual depressive symptoms may persist even after remission [66] and potentially impair global functioning [67]. Even so, research indicates high rates of nonadherence to antidepressant treatment [68,69], despite evidence supporting its efficacy [70] and effectiveness [71] compared to placebos. A study conducted among patients from the United States and five European countries revealed that antidepressant nonadherence persisted longer due to a lack of belief in the necessity for the medication [72]. Thus, ensuring that individuals who opt for antidepressant treatment are adequately informed to make informed decisions regarding their treatment and feel empowered to discuss their choices with their healthcare providers is of paramount importance.

This protocol, however, recognises potential challenges associated with the use of telehealth for mental health in regional areas. A study conducted at a regional mental health service in Australia identified several limitations of videoconferencing, including a lack of devices, unreliable internet connectivity, technical issues, privacy concerns, and online fatigue [35]. These findings are consistent with evidence from a systematic review overview that identified infrastructure- and technical-related barriers, healthcare providers’ concerns regarding increased workload and privacy, and inadequate training on digital health technologies [73]. To ensure that dyadic participants involved in the feasibility study have access to phone credit or internet to participate in the sessions, 2 GB of data will be provided to them before each SPARKED session and follow-up session. Despite issues with telehealth use, mental health service users and providers see telehealth as a valuable way to improve access to mental health care, ensure regular reviews, and support patients who face engagement challenges due to low energy, anxiety, or depression [35]. Therefore, to successfully implement telehealth in mental health services, investment in telehealth infrastructure and structured staff training is necessary to overcome identified barriers.

### 4.1. Strengths

First, this robust multi-method study will adhere to evidence-based frameworks, specifically the MRC framework [38] and the generative co-design framework [39]. The MRC framework has been chosen to guide the study’s design because of its dynamic, iterative approach to developing health interventions, while emphasising the significance of contextual factors in complex interventions [38,74]. Similarly, the generative co-design framework [39] will guide the co-design of SPARKED, thereby enhancing the study’s relevance and potential impact by ensuring it addresses real-world needs and aligns with end-user preferences. By integrating three co-design strategies, this protocol offers a systematic approach to developing a comprehensive family psychoeducational intervention aimed at improving outcomes in MDD. Second, this study harnesses the strengths of both qualitative and quantitative methodologies in developing SPARKED, as well as assessing its feasibility and acceptability. Third, this research is among the first to integrate telehealth psychoeducation for MDD through a structured PPI process, aligning with current priorities in mental health services and nursing research. By focusing on telehealth FPE, this research supports broader efforts to improve access to mental health care. It aligns with the position statement of the International Society of Psychiatric Mental Health Nurses, which recognises telehealth as an effective way to deliver mental health care and advocates for research into its utilisation for mental health services [33]. Fourth, the feasibility study phase explores the impact of the telehealth FPE on clinical and non-clinical patient-related outcomes, providing a balanced focus on outcomes that are often perceived differently by patients, their families, and healthcare providers [75]. Finally, the use of the TIDieR checklist [42] for reporting the details of SPARKED enables replication of the intervention. Although the TIDieR checklist may not fully capture contextual factors that could influence the delivery of a complex intervention [76], adherence to the MRC framework ensures this research remains grounded in identifying and reporting the research context.

### 4.2. Limitations

This protocol has some limitations. The six-week post-intervention follow-up period is relatively short, as the study’s primary aim is to assess the feasibility of the intervention and data collection methods before conducting a larger trial with a longer follow-up. As such, the study will not adequately capture the long-term effects of SPARKED. Next, the feasibility study phase focuses on patient-related outcomes. Evaluating outcomes among family members could have offered an opportunity to ascertain whether SPARKED influences outcomes for both patients and their families. In addition, the small sample size will not permit the evaluation of the intervention’s effect size. Consequently, this study cannot draw strong conclusions regarding the effects of SPARKED. A large-scale RCT of SPARKED will be required to determine the effectiveness of the intervention. Next, the exclusion criteria regarding individuals who are assessed to have imminent suicidal risks limit the generalisability of SPARKED. To improve the generalisability of this research, future studies should consider implementing strategies to optimise contacts with participants for monitoring and assessing suicide risks, as well as establishing a pathway for escalated care. Finally, using telehealth to deliver SPARKED might inadvertently exclude people who lack access to digital devices or digital skills. While this research will report all technical challenges encountered during the study (context-related) to help tailor solutions for future research, future studies should consider providing an easy-to-understand graphic guide on how to use devices or offering in-person support for participants to address this limitation.

### 4.3. Conclusions

This nurse-led study will involve patients with MDD, their family members or significant others, and mental healthcare providers in co-designing telehealth FPE for MDD aimed at improving patient-related outcomes. This research will further assess the feasibility and acceptability of the intervention while examining its preliminary impact on both clinical and non-clinical outcomes. Nurses play a crucial role in mental health care and are uniquely positioned to support patients with MDD throughout their recovery journey. Given that nursing care extends beyond patient care to encompass the support of patients’ family members and social networks, the implementation of FPE by nurses can be effectively embedded within their established professional roles and routine clinical practice. Nursing educational programs and training initiatives should aim to empower nurses to lead innovations that expand nursing care, with the goal of improving patient outcomes and experiences. More importantly, viable policies and strong nursing leadership are essential for creating environments that support evidence-based practices, health innovations, and systemic change in response to the constantly evolving healthcare landscape.

## Figures and Tables

**Table 1:** Key features of the SPARKED protocol.

Element	SPARKED Protocol
Type of intervention	Family psychoeducation
Study participants	Patients with MDD and family members, carers or significant others
Mode of delivery	Telephone or videoconferencing
Format	Single-family
Number of SPARKED sessions	Three
Frequency of sessions	Bi-weekly
Session duration	45–60 min
Intervention duration	Six weeks
Follow-up	At Week 12 (six-week post-intervention)
Outcome measurement timepoints	At baseline, immediately after intervention, and at follow-up

## Data Availability

No new data were created or analysed in this study. Data sharing is not applicable to this article.

## Supplementary Materials

The following supporting information can be downloaded at https://www.mdpi.com/article/10.3390/nursrep15100364/s1, Table S1: Preliminary SPARKED guide; Table S2: Semi-structured interview guide: co-designing telehealth family psychoeducational intervention for major depressive disorder with family dyads; Table S3: Mental health professionals’ survey: co-designing telehealth family psychoeducational intervention for major depressive disorder; Table S4: Semi-structured interview guide: assessing retrospective acceptability of SPARKED among patients with major depressive disorder and their family members or significant others.

## Public Involvement Statement

No members of the public were involved in the design of this study protocol; however, people with lived experience of MDD and their family members or carers will be closely involved in the intervention co-design.

## Guidelines and Standards Statement

This protocol is designed according to the TIDieR (Template for Intervention Description and Replication) guidelines for reporting intervention development.

## Abbreviations

The following abbreviations are used in this manuscript:

FPE	Family psychoeducation
MDD	Major depressive disorder
MRC	Medical Research Council
SPARKED	Supportive Program for Advancing Recovery, Knowledge, and Empowerment in Depression
SSI	Semi-structured interview
