# Supplementary material for: Telehealth Family Psychoeducation for Major Depressive Disorder: A Protocol for Intervention Co-Design and Feasibility Study
Source: Nurs Rep. 2025 Oct 11;15(10):364. doi: 10.3390/nursrep15100364 (PMC12566970; doi:10.3390/nursrep15100364)
Supplement: Supplementary file 1 [file nursrep-15-00364-s001.zip › nursrep-3873137-supplementary.pdf]

Supplementary File

Table S1: Preliminary SPARKED guide

**Session and Tentative Topics**
**Session 1: Major depressive disorder**<br>• Course and causes<br>• Symptomology and impact<br>• Evidence-based treatments and rationales<br>• Misconceptions and stigma<br>• Risks<br>• Exploring perceived treatment necessity and concerns<br>• Exploring participants-defined outcomes
**Session 2: Self-efficacy and self-assertiveness**<br>• Coping skills: Person with MDD and support person<br>• Developing personal recovery-focused approach<br>• Being an active participant in treatment planning<br>• Quality of life: Person with MDD and support person<br>• Exploring participants' recovery journey
**Session 3: Relapse prevention**<br>• Optimising lifestyle modifications<br>• Identifying signs of relapse or recurrence<br>• Developing an action plan<br>• Exploring participants-defined outcomes
**6-week follow-up session: Review of the following topics**<br>• Course of illness and risks<br>• Personal recovery-focused approach<br>• Semi-structured interview assessing retrospective acceptability

Table S2: Semi-structured interview guide – Co-designing telehealth family psychoeducational intervention for major depressive disorder with family dyads

Ques. No.	Opening Question	Probing Questions
1.	How long in years have you been diagnosed with depression? 1 year, 2, 3, 4, or more?	• Can you tell me where the diagnosis was made?
2.	Can you tell me the type of treatment for depression you have received?	• What treatment are you currently taking to treat depression?
3.	Can you share your experiences of the treatment strategy that has been helpful for you to manage depression or support your family member with depression?	• How did you know about the strategy?<br>• Are you currently using the strategy?
4.	What is the most helpful information and support you received from health professionals, for example, a GP, psychiatrist, nurse, or psychologist, regarding depression and your treatment?	• Can you share how this information or support made an impact on your health / your relative's health?
5.	Can you share your experiences of challenges you might have faced while seeking treatment or support to manage depression?<br><br>Can you share your experiences of challenges you might have faced while seeking depression treatment for your relative?	• Do the challenges still exist?<br>• If yes, what do you think the health professionals could do to address the challenge?<br>• If no, how did you address the challenge?
6.	How do you feel about the information on depression and your treatment provided by your GP, psychiatrist, nurse, or psychologist?	• Can you tell me why you feel so?
7.	What kind of information would you like healthcare professionals involved in depression care to provide to patients living with MDD and their families?	• Why do you need this information?<br>• How important is this information to your treatment?
8.	Apart from seeking information about depression, treatment, and self-management skills, are there other problems you would like the health professionals to support you with?	• Can you tell me how this problem affects your mental health?

9.	Have you ever received telehealth services from your healthcare provider?	• If no, how do you view the telehealth service?<br>• If yes, what was the telehealth service, and for what purpose?<br>• How did you feel about the telehealth service?
10.	What are your opinions about telehealth family psychoeducation for depression?	• Would you consider accessing telehealth family psychoeducation in depression?<br>• Why or why not?
11.	How often would you like to receive telehealth family psychoeducation sessions from health professionals involved in your depression care?	• What telehealth method do you prefer: telephone or videoconferencing?<br>• How long would you prefer a family psychoeducation session last?

Table S3: Mental health professionals' survey – Co-designing telehealth family psychoeducational intervention for major depressive disorder

This survey gathers your views on the information needs of families and patients with major depressive disorder (MDD) and on telehealth family psychoeducation to improve treatment outcomes in MDD. It should take no more than 10 minutes to complete.

By clicking the "Next" button, you agree that you have read and understood the information on the consent form, had the opportunity to ask any questions about the study, and consented to participate in this survey.

**Section 1: Professional demographics**	
a. What is your clinical position at your workplace (mental health setting)?	☐Clinical nurse specialist<br>☐Nurse practitioner<br>☐Occupational therapist<br>☐Psychiatric registrar<br>☐Psychiatrist<br>☐Psychologist<br>☐Registered nurse<br>☐Social worker<br>☐Other (Please specify): ……………………………………..
b. How many years have you been practising as a mental health professional?	☐Less than 3 years<br>☐3 – 5 years<br>☐6 – 10 years<br>☐11 – 15 years<br>☐More than 15 years
c. What is your employment status in your current clinical position?<br>**Select an option that applies.**	☐Permanent full-time<br>☐Permanent part-time<br>☐Fixed-term contract<br>☐Other (Please specify): …………………………………..
d. What is the highest professional qualification you have achieved that is relevant to your role in mental health?<br>**Select an option that applies.**	☐Bachelor's degree<br>☐Graduate certificate<br>☐Graduate diploma<br>☐Master's degree<br>☐Doctoral degree<br>☐Residency program
e. Which of the following best represents your gender?	☐Woman<br>☐Man<br>☐Non-binary<br>☐Prefer not to say<br>☐Other (You can specify): …………………………………..

f.	Which best describes your overall duties in your current mental health practice?<br><br>**Select all that apply.**	☐Clinical assessment<br>☐Diagnosing<br>☐Care or treatment planning<br>☐Case management<br>☐Follow-up and monitoring<br>☐Other (Please specify): ……………………………………..

Section 2: Your experience working with patients with major depressive disorder (MDD)

a.	In your mental health professional role, how often do you find MDD patients supported by a family member or significant others to seek treatment?	☐Never<br>☐Rarely<br>☐Sometimes<br>☐Often<br>☐Always
b.	What information should health professionals provide to MDD patients and their family members or significant others to facilitate recovery from the disorder?<br>**Select all that apply.**	**Information related to:**<br>☐ Major depressive disorder<br>☐ Treatment options<br>☐Antidepressant medication effects<br>☐Illness management skills<br>☐Family support<br>☐Services to address social stressors, such as accommodation problems.<br>☐Other topics: ………………………………………
c.	What information do MDD patients and their family members or significant others seek from health professionals who are involved in depression care or treatment?	Please specify:<br>…………………………………………………………………<br>…………………………………………………………………<br>…………………………………………………………………

Section 3: Your perspective on telehealth family psychoeducation for patients with MDD and their families or significant others.

a.	What do you think is essential for effective **<u>telehealth</u>** family psychoeducation for MDD?	Please specify:<br>…………………………………………………………………<br>…………………………………………………………………..

		Yes	Uncertain	No
b. Do you have concerns about **telehealth** family psychoeducation for patients with MDD and their family members or significant others?<br><br>**Select the options that describe whether you have concerns.**	May pose a limitation to clinical risk management			
	Technical difficulties			
	Privacy and security risks			
	Accessibility issues regarding internet access or phone data			
	Loss of non-verbal communication with telephone care delivery			
	Other issues: …………………………………….			
c. What do you think about the potential impact of **telehealth** family psychoeducation on outcomes in MDD?	☐Improved outcomes<br>☐No change<br>☐Worsened outcomes			

Table S4: Semi-structured interview guide – Assessing retrospective acceptability of SPARKED among patients and their family members or significant others

	Family ID:	Individual with MDD ☐ Family Member ☐
	**Question domain**	**Opening and Probing Questions**
1	General thoughts about SPARKED	What are your thoughts about the telehealth family psychoeducation (FPE) intervention used in this research?<br>**Probing questions:**<br>• Can you tell me more about your thoughts on the intervention?<br>• How do you think the FPE sessions worked in terms of enhancing your knowledge of MDD and empowering you towards recovery?
2	FPE contents of SPARKED	How did you feel about the topics discussed during the FPE sessions?<br>**Probing questions:**<br>• What FPE topics do you view as important to you and your family?<br>• What FPE topics do you view as less important to you and your family?
3	Facilitators of SPARKED	Are there factors that you think contributed to the success of the FPE (intervention)?<br>**Probing questions:**<br>• Considering the delivery of the FPE session, were there things that specifically helped to improve delivery?<br>• Considering the follow-up, were there things that specifically helped to improve the follow-up?
4	Barriers encountered	Are there factors that you think contributed to the FPE (intervention) not working well?<br>**Probing questions:**<br>Considering the delivery of the FPE session, were there things that specifically did not help to improve the delivery?<br>Considering the follow-up, were there any specific things that did not help improve the follow-up?
5	Suggestions for SPARKED improvement	Are there things that could have been done to improve the family psychoeducational sessions?<br>**Probing questions:**<br>What would you suggest for an improvement on the FPE used in this research?
6	Explore any other issues	Finally, are there any issues that we haven't discussed during this interview that you would like to bring up?
